# Ion Channels and Pumps in Autophagy: A Reciprocal Relationship

**DOI:** 10.3390/cells10123537

**Published:** 2021-12-14

**Authors:** Hussein Abuammar, Arindam Bhattacharjee, Zsófia Simon-Vecsei, András Blastyák, Gábor Csordás, Tibor Páli, Gábor Juhász

**Affiliations:** 1 Lysosomal Degradation Research Group, Institute of Genetics, Biological Research Centre, 6726 Szeged, Hungary; abuammar.hussein@brc.hu (H.A.); bhattacharjee.arindam@brc.hu (A.B.); blastyak.andras@brc.hu (A.B.); csordas.gabor@brc.hu (G.C.); 2 Department of Biology, Faculty of Science and Informatics, University of Szeged Doctoral School, 6726 Szeged, Hungary; 3 Department of Anatomy, Cell and Developmental Biology, Faculty of Science, Eötvös Loránd University, 1117 Budapest, Hungary; simon.vecsei.zsofia@ttk.elte.hu; 4 Membrane Biophysics Research Group, Institute of Biophysics, Biological Research Centre, 6726 Szeged, Hungary; pali.tibor@brc.hu

**Keywords:** autophagy, ion channels, V-ATPase, TRPML, calcium

## Abstract

Autophagy, the process of cellular self-degradation, is intrinsically tied to the degradative function of the lysosome. Several diseases have been linked to lysosomal degradative defects, including rare lysosomal storage disorders and neurodegenerative diseases. Ion channels and pumps play a major regulatory role in autophagy. Importantly, calcium signaling produced by TRPML1 (transient receptor potential cation channel, mucolipin subfamily) has been shown to regulate autophagic progression through biogenesis of autophagic-lysosomal organelles, activation of mTORC1 (mechanistic target of rapamycin complex 1) and degradation of autophagic cargo. ER calcium channels such as IP_3_Rs supply calcium for the lysosome, and lysosomal function is severely disrupted in the absence of lysosomal calcium replenishment by the ER. TRPML1 function is also regulated by LC3 (microtubule-associated protein light chain 3) and mTORC1, two critical components of the autophagic network. Here we provide an overview of the current knowledge about ion channels and pumps—including lysosomal V-ATPase (vacuolar proton-ATPase), which is required for acidification and hence proper enzymatic activity of lysosomal hydrolases—in the regulation of autophagy, and discuss how functional impairment of some of these leads to diseases.

## 1. Introduction

Autophagy is a catabolic process that efficiently degrades cellular cargo, including but not limited to organelles, membranes, nuclear material and pathogens within lysosomes [1]. While the first *Atg* genes were discovered in the 1990s, the regulation of the process, especially the last degradative step inside the lysosome, is only recently beginning to receive more attention. All forms of autophagy, namely, macro- and microautophagy and chaperone-mediated autophagy, rely on lysosomal function. The mechanisms of these latter processes were covered in recent reviews [2,3]; therefore, in this review we focus on the role of cellular ion homeostasis in regulation of macroautophagy (autophagy hereafter). Briefly, the process starts by nucleation of so-called “phagophores” from multiple cellular membrane sources, such as the ER. Phagophore elongation is promoted by the interplay of ubiquitin-like conjugation pathways that result in covalent conjugation of LC3-family proteins to PE in the double-membrane phagophore, which is an important molecular step in the autophagic process. The phagophore engulfs cargo targeted for degradation either in non-selective (“bulk”) or selective manner, the latter utilizing selective autophagy receptor proteins [4]. The sealed autophagosome finally attains maturity by recruitment of SNARE proteins and small GTPases, including Rab7, to its outer membrane [5,6]. This is accompanied by the activation of lysosomes and their movement along microtubules for the purposes of fusion with the help of multiple microtubule-interacting partners, such as the small GTPase Arl8 and the calcium binding protein ALG-2. [7,8]. We and others have shown that autophagosome-lysosome fusion occurs via Rab7-mediated assembly of membrane tethers (e.g., the HOPS complex) to link the two organelles, and a fusion step orchestrated by dedicated SNARE proteins (e.g., syntaxin 17 and/or Ykt6 on autophagosomes, VAMP7/8 on lysosomes and SNAP29 in-between these) [5,9,10,11]. After fusion, the cargo is degraded in the highly acidic (pH 4.5–5) lumens of the autolysosomes by the action of acidic hydrolases. The entire flow of materials from phagophore biogenesis to eventual fusion and degradation is referred to as “autophagic flux,” the impairment of which has been documented in numerous diseases ranging from proteinopathies to rare lysosomal storage disorders.

Lysosomal degradation is regulated by the integrated inputs of multiple upstream mechanisms, key among which is the mTOR pathway. mTOR is a serine/threonine kinase acting as a master regulator of protein synthesis and cell proliferation. The two multiprotein complexes, namely, mTORC1 and mTORC2, differ in subunit composition, downstream targets and sensitivity to rapamycin inhibition [12]. mTORC1 is composed of three core components: mTOR, mLST8 and a specific subunit, the scaffold protein raptor. This complex integrates information about environmental status and nutritional abundance to coordinate the balance between anabolism and catabolism inside the cell. mTORC2 is built of mTOR and mLST8; it similar to mTORC1 but contains the scaffold protein rictor. This complex regulates cytoskeletal rearrangements and activates pro-survival and proliferation pathways. While mTORC1 can be acutely inhibited by rapamycin, mTORC2 responds only to prolonged rapamycin treatment [12]. Rheb and Rag GTPases are regulators of mTORC1 that act by changing its activity and localization, respectively. Rheb stimulates mTORC1 kinase activity when the cellular environment is abundant in growth signals and nutrients [13]. Additionally, a Rag heterodimer recruits mTORC1 from the cytosol to the surface of the lysosome if glucose, amino acids and other nutrients are available inside the cell [14]. Of note, the Ragulator complex was shown to be required for the lysosomal localization of mTORC1 as well, acting as a scaffold protein for Rags [14].

As described above, mTORC1 activation can happen only if intra- and extracellular conditions support sustained growth, and hence mTORC1 can upregulate protein, lipid and nucleotide synthesis pathways. In line with this, mTORC1 signaling negatively regulates autophagy by inhibiting ULK1 and ATG13 through their phosphorylation [15]. Upon mTOR inactivation during amino acid withdrawal, active ULK1 kinase complex phosphorylates the Vps34 complex subunit Beclin1. Interaction of phosphorylated Beclin1 with Atg14L1 in the Vps34 complex enhances the class III phosphatidylinositol 3-phosphate kinase (PI3K-III) activity of Vps34, leading to recruitment of PI3P effectors WIPI2 and DFCP1 to promote phagophore formation at PI3P-rich ER subdomains called omegasomes [16,17,18].

While mTORC1 is an important upstream regulator of the cellular degradation machinery, the downstream factor that is responsible for lysosomal acidification and therefore effective completion of autophagy, the multi-subunit V-ATPase complex, is equally important. Furthermore, lysosomes are a secondary reservoir of calcium ions in the cell. They stay close to ER Ca^2+^ exit sites, especially IP_3_R, which delivers ER calcium to lysosomes [19,20]. Lysosomal Ca^2+^ efflux channels such as mucolipin 1/TRPML and TPC2 have been shown to be involved in multiple steps in the autophagy–lysosomal pathway, such as altering the pH and therefore the degradative function of lysosomes; the biogenesis of autophagy and lysosomal components; and regulation of the cellular nutrient sensor mTORC1 [21,22]. Here we summarize the recent advances in the roles of ion channels and ion pumps in the regulation of autophagy and lysosomal function.

## 2. Ion Channels and Autophagy

The ionic content of the lysosome lumen is markedly distinct from that of the cytosol. The concentrations of Na^+^, Ca^2+^ and H^+^ are higher and the K^+^ concentration is lower inside lysosomes [23,24,25,26,27]. This unequal distribution of ions creates a slight lumen-positive membrane potential that is maintained by the action of lysosomal ion channels [28]. The primary source of lysosomal Ca^2+^ is the endoplasmic reticulum; thus, non-lysosomal ion channels are also important for maintaining lysosomal ion homeostasis. Below, we discuss the role of those ion channels, whose function appears to be involved in various steps of autophagy, such as mTOR activation, biogenesis of autophagosomes and their fusion to lysosomes and lysosomal cargo degradation. Wherever appropriate, holding to the aims of this review, the involvement of the given channel in pathologies is also mentioned.

### 2.1. K^+^ Channels

Perturbation of K^+^ levels can induce bulk autophagy [29]. K^+^ deprivation was found to be a potent inducer of autophagy utilizing the core autophagy machinery components Atg1, Atg5 and Vps34 kinase in *Saccharomyces cerevisiae*. This is thought to occur in response to changes in cell volume, pH and turgor pressure in the absence of K^+^ [29]. These observations are in concert with the fundamental role of autophagy upon perturbation of ion homeostasis, but by no means indicate a direct regulatory role of potassium. However, this conclusion may need to be refined by studies addressing the role of plasma membrane potassium channels in tumor progression. Both normal and metastatic tissues employ voltage-gated potassium channels to regulate cell proliferation [30,31]. Targeting of Kv11.3, a plasma membrane voltage-gated K^+^ channel, has been used to inhibit proliferation and activate senescence of breast cancer cells [32]. Furthermore, high expression of Kv11.3 or using its pharmaceutical activator NS1643 was also shown to control breast cancer metastasis [33]. A possible molecular mechanism for this regulation was proposed, where the authors showed that Kv11.3 plays a crucial part in regulating autophagic flux through AMPK activation [34]. Under glucose starvation conditions, active AMPK phosphorylates ULK1 at Ser317 and Ser777 for its autophagic activation. Notably, mTORC1 disrupts this AMPK-ULK1 interaction by ULK1 phosphorylation at Ser757 upon glucose sufficiency [15]. The authors speculate that this AMPK-induced autophagic activity is able to drive cells towards senescence to thwart apoptotic cell death. Autophagy inhibition via siRNA targeting of AMPK or hydroxychloroquine, an autophagy inhibitor, was found to induce apoptosis in NS1643-treated cells. Therefore, autophagy modulation was postulated as a therapeutic route to prevent cell survival by autophagic senescence [34].

Another plasma membrane K^+^ channel, hERG1 encoded by ether-à-go-go related gene 1, is expressed in a wide range of human carcinomas (breast, endometrial, ovarian, pancreatic, esophageal, stomach and colorectal) [35]. hERG1 interacts with PI3K, which through PDK1, activates Akt1. Phospho-Akt1 indirectly activates mTOR function, thereby inhibiting autophagy induction and promoting cell survival. Therefore, hERG1-expressing colorectal cancer cells are resistant to chemotherapy. Clarithromycin (CLA), a specific inhibitor of hERG1, preferentially binds to the closed conformation of this ion channel to inhibit hERG1-PI3K complex formation, and therefore reduces Akt phosphorylation, leading to increased autophagic flux. However, prolonged hERG1 blockage by CLA was shown to block autophagic flux and render colorectal cancer cells more susceptible to 5-fluorouracil [36].

The most convincing evidence for a direct regulatory role of potassium in maintaining lysosomal function comes from the analysis of *TMEM175*. *TMEM175* encodes a major endo-lysosomal K^+^-selective channel K_EL_ that contributes to lysosomal membrane potential and pH_[lys]_. Mutations in this channel were reported to impair autophagosome-lysosome fusion [37]. *Tmem175* KO causes abnormal pH_[lys]_, which leads to impaired mitochondrial respiration, an autophagosome clearance defect, decreased glucocerebrosidase activity and increased α-synuclein aggregation [38].

### 2.2. Ca^2+^ Channels

**Figure 1:**
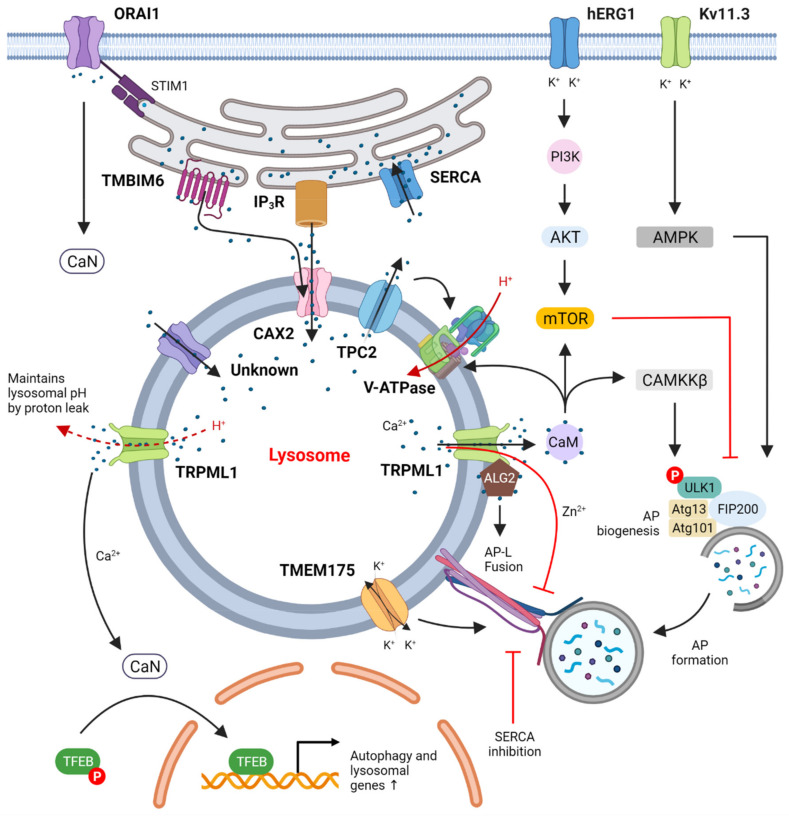
Suggested regulation of autophagy by ion channels. Calcium channels in the plasma membrane, such as ORAI1 (together with its ER binding partner STIM1), take up extracellular Ca^2+^, activate calcineurin (CaN) and thus lead to dephosphorylation of TFEB. Plasma membrane K^+^ channels such as hERG1 and Kv11.3 that take up extracytosolic K^+^ were shown to regulate the PI3K/Akt/mTOR and AMPK/ULK1 pathways, respectively, to control autophagy. ER Ca^2+^ channels such as SERCA pump cytosolic Ca^2+^ into the ER lumen while IP_3_R and TMBIM6 transfer Ca^2+^ from ER into the lysosomes at ER-lysosome membrane contact sites through the lysosomal Ca^2+^ importer CAX2 and other yet-unidentified calcium importers. These actions together maintain lysosomal Ca^2+^ homeostasis, which appears to be critical for autophagic degradation and autophagic lysosome reformation. Lysosomal Ca^2+^ channels such as TPC2 and TRPML1 release lysosomal Ca^2+^ to the cytosol to activate the CaM/CAMKKβ and CaN/TFEB pathways that increase autophagic flux via both transcription-independent and transcription-dependent mechanisms, respectively. The only known lysosomal proton pump, V-ATPase, pumps H^+^ into lysosomes to maintain acidification and is regulated by CaM binding to V0 in neurons.

Elevation of cytosolic Ca^2+^ is known to trigger autophagy [39], although the physiological source of Ca^2+^ ions during autophagy remains ambiguous. Firstly, in a well-characterized pathway in both yeast and mammals, direct sensing of abnormal ER luminal Ca^2+^ (for example, due to pharmacological blockage of SERCA) is detected by ER resident proteins that are involved in the unfolded protein response (UPR). In mammals, three parallel UPR pathways are responsible for restoring ER protein folding capacity by transcription of chaperone proteins and activating cytoprotective autophagy [40,41,42]. Next, calcium-induced autophagic activation is dependent on core Atg proteins and CaMKKβ signaling—inhibition of Atg7 and CaMKKβ results in fewer autophagosomes produced after an elevation in Ca^2+^_[cyt]_ by Ca^2+^-mobilizing agents TG and ATP [43]. ER-localized Bcl-2 (best known as an anti-apoptotic protein) can interfere with the mobilization of ER calcium, and therefore inhibit autophagy. The mechanism has been shown to depend on regulation of AMPK activity by CaMKKβ, which in turn inactivates mTORC1 to inhibit cell growth and promote autophagy (Figure 1). In another study, exogenously introduced calcium in the form of CPP activated, de novo, synthesis of autophagosomes mediated by core autophagy machinery components such as Beclin1, FIP200 and several other Atg proteins from the LC3 conjugation cascade. CPP induced the formation of punctate DFCP1 structures associated with PI3P-enriched subdomains (also known as omegasomes) of ER where autophagosomes can form [44,45]. Intracellular surge of Ca^2+^ alone can also increase mTOR activity, which can be mimicked by calcium ionophores TG and ionomycin. This atypical mTOR activation can be reversed by inhibition of either the lysosomal V-ATPase, the key to lysosome acidification, or RagC, a small GTPase and mTOR activator on the lysosome. This indicates that the response is dependent on V-ATPase and its associated protein complex Ragulator that binds mTOR [46]. In general, ER calcium plays pivotal and highly context-dependent roles for both positive and negative regulation of autophagy [41,47].

#### 2.2.1. Sarco/Endoplasmic Ca^2+^ ATPase

SERCA is a sarcoplasmic and ER-based, ATP hydrolysis-dependent Ca^2+^ influx channel required for calcium accumulation in ER. The essential role of the ER in general (the largest intracellular calcium store) and of Ca^2+^_[cyt]_ in autophagy progression, are highlighted by the fact that Ca^2+^ dyshomeostasis triggered by the calcium ionophore A23187 and the SERCA inhibitor TG could block autophagy by inhibiting autophagosome expansion and closure [48]. Mechanistically, local calcium regulation in the ER-phagophore contact areas and their close physical association were found to be orchestrated by SERCA and VMP1. Thus, SERCA inhibition by TG likely stalls phagophore formation at ER contact sites, and therefore prevents functional autophagy [49]. ER calcium depletion leads to a toxic/misfolded protein load in the ER lumen that mounts UPR through three distinct ER-localized proteins—IRE1, ATF6 and PERK—and distinct downstream factors that eventually aid in the transcription of ER chaperones [50]. SERCA inhibitors have been shown to activate UPR by depleting Ca^2+^_[ER]_, which also activates pro-death autophagy by the CAMKK-AMPK-mTOR axis [51,52]. Furthermore, influenza A infection was shown to block autophagosome-lysosome fusion in host cells through inhibition of SERCA by AM2. This way, the virus can induce cell death to regulate its proliferation and antigenic responses [53]. Pharmacological activation of SERCA in influenza A-infected cells was found to accelerate autophagic fusion and degradation, and thus reduce the inflammatory burden on host tissue [54]. SERCA-mediated calcium homeostasis also plays an important role in degradation of autophagic material. SERCA inhibition by bafilomycin A1 increases Ca^2+^_[cyt]_, which attenuates autophagosome-lysosome fusion in *Drosophila* [55]. Specifically, SERCA inhibition can impact ER-localized Ca^2+^ pools, which likely provide the Ca^2+^ gradients necessary for vesicle fusion by SNARE proteins and associated factors.

#### 2.2.2. Inositol Triphosphate Receptor Ca^2+^ Channel

ER luminal calcium and ER calcium channels are necessary for refilling lysosomes with calcium after lysosomal Ca^2+^ release. Low pH_[lys]_ is necessary to maintain the ER-lysosome contact sites where Ca^2+^ transport takes place via IP_3_R and a putative lysosomal Ca^2+^ importer [19]. Further, maintaining homeostatic levels of Ca^2+^_[lys]_ is necessary for lysosomal degradative activity [56]. The Ca^2+^ efflux channel IP_3_R localizes to ER and has been shown to regulate ER-mitochondrial and ER-lysosomal calcium transport, both of which may be important for autophagosome formation [41]. Firstly, steady low-level ER-mitochondrial calcium transport is important for mitochondrial bioenergetics and ATP production [57]. In support of this, upregulation of LC3 mRNA levels was observed with IP_3_R overactivation in a Duchenne muscular dystrophy mouse model; inhibition of IP_3_R was able to restore basal autophagic levels [58]. Failure in transporting Ca^2+^ ions to the mitochondria, for example, due to the blocking of IP_3_R, activates the AMPK-ULK1 axis to stimulate pro-survival autophagy [59]. Evidence for the context-dependent role of ER-mitochondrial Ca^2+^ transport in autophagy also comes from disrupting the interaction between ER Ca^2+^ channel IP_3_R and mitochondrial-resident VDAC1 by their respective organelle tethers GRP75 and TOM70. In the absence of this critical ER-mitochondrial Ca^2+^ transport, pro-death autophagy by cadmium (a neurotoxin) was inhibited in rat primary neurons; and surprisingly, pro-survival autophagy was found elevated in mouse embryonic fibroblasts via AMPK activation [60,61]. An interesting regulatory role of IP_3_R was highlighted during the pathogenesis of a mouse non-alcoholic fatty liver disease (NAFLD) model established using high oleic acid diet. In this model, gradual hyperactivation of IP_3_R was observed, which eventually led to excessive Ca^2+^_[cyt]_ and Ca^2+^-dependent activation of ERK1/2, ultimately leading to downregulation of autophagy via mTOR and FOXO3 activation. As a result, lipid cargo in hepatocytes was not degraded, leading to lesions [62]. Furthermore, in cells where ER-lysosome Ca^2+^ transport was inhibited by the IP_3_R inhibitor xestospongin, the lysosomes had aberrant morphology and were not degrading cargo properly, resembling lysosomal storage disorders [20]. In summary, depleted ER luminal Ca^2+^, and Ca^2+^ transients from IP_3_R are sufficient to induce autophagy, although the mechanisms are different (in general, unfolded protein response in case of luminal Ca^2+^ depletion, and mitochondrial impairment in case of IP_3_R blockage) [41].

#### 2.2.3. Transient Receptor Potential Mucolipin Channels (TRPMLs)

Mammalian TRPML1–3 are tetrameric non-selective cation channels with six transmembrane domains, which localize exclusively on endosomes and lysosomes. They are permeable to Ca^2+^, Na^+^ and heavy metal ions (such as Fe^2+^ and Zn^2+^), and they also permeate K^+^. TRPML1, a non-selective lysosomal cation channel, is associated with the lipid storage disorder mucolipidosis type IV (MLIV) characterized by accumulation of amphiphilic lipids in large endolysosomal compartments (See Section 5). TRPML1 mediates lysosomal calcium efflux and is essential during starvation-induced autophagy [63,64]. Long, but not short periods of serum starvation (4 h) result in substantial TRPML1 Ca^2+^ current, which can be mimicked by the mTOR inhibitor torin-1 [65]. Highlighting its regulatory potential, TRPML1 itself is a phosphorylation target of lysosomal mTOR. During amino acid (AA) starvation, TRPML1 phospho-inhibition is reversed due to mTOR detachment from lysosomes, leading to Ca^2+^ efflux. Juxtalysosomal Ca^2+^ can bind to CaM and promote lysosomal mTOR recruitment during prolonged starvation to restore growth signaling [66]. Thus, the TRPML1 phospho-switch might play an important role in orchestrating autophagy-lysosomal responses to nutrient starvation. Intracellular Ca^2+^ entry in response to AA abundance has also been shown to activate mTOR via CaM’s interaction with TSC2, a negative regulator of mTOR, although the AA-sensitive Ca^2+^ channels involved in the process remain elusive [67]. The connection between cytosolic calcium and mTOR activity has been extensively discussed. Treatment with the Ca^2+^ chelator BAPTA abolishes rapamycin-induced autophagy, indicating the importance of cytosolic Ca^2+^ in the canonical mTOR-dependent autophagy pathway [68]. Further investigation showed that calcium release by TRPML1 has a regulatory role in mTOR activation. TRPML1 depletion by lentiviral shRNA inhibits mTORC1 activity in HEK293T cells, which is due to the blocking of lysosomal Ca^2+^ release. Overexpression of TRPML1 or treatment with the TRPLM agonist MLSA1 has the opposite effect: the level of phosphorylated S6K, an mTORC1 substrate, was increased. Additionally, the activating effect of Ca^2+^ seemed to be CaM dependent, which is strengthened by the finding that CaM and mTOR can directly interact with each other [69]. Notably, TRPML1 itself is a phosphorylation target of lysosomal mTOR, and after phosphorylation, the activity of the ion channel is decreased. This feedback regulation of mTOR likely plays a crucial role in cellular homeostasis during stress [66].

TRPML1 was also implicated in the biogenesis of autophagic structures in both transcription-dependent and independent manners: First, TRPML1 calcium-dependent activation of the calcium-regulated phosphatase CaN results in dephosphorylation of lysosomal resident TFEB, thereby triggering its release from the negative regulator 14-3-3 and subsequent nuclear translocation to promote the biogenesis of autophagy and lysosomal components [70]. Second, a recent study showed that rapid calcium efflux by TRPML1 is able to activate the calcium-dependent kinase CaMKKβ and downstream AMPK pathways, which upregulate ULK1 and VPS34 autophagic complexes to trigger phagophore formation [21]. Intriguingly, *Drosophila Trpml* mutants demonstrated suppression of TOR kinase and elevated basal autophagy. This apparent contradiction was suggested to be due to impaired fusion and degradation of existing autophagosomes in *Trpml* mutants, and thus reduced overall cellular AA availability, which could inactivate mTOR [71]. Thus, TRPML1-dependent Ca^2+^ efflux from lysosomes may regulate autophagy depending on the duration and scale of its activation.

It was also suggested that TRPML1 channels play roles in autophagosome–lysosome fusion. AA deprivation activates TRPML1 Ca^2+^ release, which is sensed by ALG2. Ca^2+^-activated ALG2 binds to the dynactin-dynein motor protein complex, driving the lysosomes towards the perinuclear region where they fuse with autophagosomes [8]. Indeed, a pharmaceutical activator of TRPMLs increased autophagosome-lysosome fusion, and the opposite effect was seen upon inhibition of microtubule assembly by vinblastine or by silencing the autophagic SNARE factor, syntaxin 17 [21]. TRPML1 might also act as a negative regulator of autophagosome-lysosome fusion, as it was found to inhibit autophagy by mediating lysosomal zinc efflux in pancreatic cancer cells. Zn^2+^ released by TRPML1 was recently shown to inhibit the interaction between autophagosomal syntaxin 17 and lysosomal VAMP8 SNAREs, thereby blocking autophagosome-lysosome fusion [72].

TRPML1 may also play an important role in lysosomal degradation, as autophagy is defective in TRPML1-deficient motor neurons. A similar phenotype was observed in a mouse ALS model where motor neurons were exposed to the neurotoxin L-BMAA. In both cases, autophagic degradation was impaired, leading to lipidated, autophagosome-associated LC3-II and p62 accumulation, eventually promoting neurotoxicity. The selective TRPMLs agonist ML-SA1 was able to restore normal autophagic flux in L-BMAA-exposed neurons and suppress cell death [73,74]. TRPML1 functions as a sensor for ROS that originate from damaged mitochondria. In response to this stimulus, TRPML1-initiated Ca^2+^ signaling activates CaN-dependent nuclear translocation of TFEB for lysosomal biogenesis and autophagy induction, leading to clearance of damaged mitochondria [75]. Further highlighting the importance of TRPML1-mediated Ca^2+^ release in the maintenance of autophagic flux, a novel estradiol-derived specific TRPML1 antagonist EDME was shown to effectively block autophagic degradation and cell migration in estrogen-receptor negative cancer cells [76].

Notably, TRPML has been implicated in regulating lysosomal pH [77]. Lysosomes in MLIV patient-derived cells are highly acidic because they fail to maintain resting pH_[lys]_ via the proton leaking activity of TRPML. These highly acidic lysosomes are unable to degrade autophagic cargo (mostly membrane and lipids because too low of a pH_[lys]_ impairs lipid hydrolysis), leading to lipid storage disorder phenotypes [77]. TRPML is activated by PI(3,5)P2, a membrane lipid generated from PI(3)P by the action of PIKfyve kinase. In *PIKfyve*-silenced cells, TRPML remains unphosphorylated, so its Ca^2+^ efflux activity is inhibited, leading to impaired acidification. Importantly, ML-SA1 treatment of *PIKfyve*-silenced cells could rapidly reacidify lysosomes [78]. Regulation of pH_[lys]_ by lysosomal calcium flux thus has a key role in promoting proper lysosomal enzyme activity.

#### 2.2.4. Two-Pore Channels TPC1 and TPC2

NAADP is a second messenger that can effectively mobilize Ca^2+^ from acidic stores, such as the lysosome. Key lysosomal effectors of NAADP are the Ca^2+^ efflux two-pore channels (TPCs), TPC1 and TPC2, which together with a putative Ca^2+^/H^+^ exchanger form a resting lysosomal calcium and proton gradient. TPCs are localized exclusively on endosomes and lysosomes and are ubiquitously expressed in mammalian tissues. As their name suggests, TPCs contain two putative pore loops between 12 transmembrane domains and are gated by NAADP. Interestingly, apart from NAADP, TPC2 Ca^2+^ transients are also observed during mTOR loss-of-function, for example, during autophagy or rapamycin treatment [79]. Concomitantly, *Tpc* mutant cells display elevated mTOR activity and thus inability to sense limiting cell density during in vitro growth conditions [80]. It has been speculated that TPC Ca^2+^ efflux blocks autophagosome-lysosome fusion by raising pH_[lys]_, although the exact mechanisms are unclear [81].

TPCs have been implicated in trafficking of virus particles and bacterial toxins [82,83]. More recently, TPC2 function was shown to be linked to lysosomal degradation. Contrasting evidence points to TPC regulation of lysosomal pH, and by extension its degradative properties—*Tpc2^−/−^* mouse myoblasts display reduced mTOR activity and thus exacerbated autophagic flux. *Tpc2* mutants also show reduced lysosomal protease activity and elevated pH_[lys]_, cumulatively leading to accumulation of autophagic markers [22]. In another study, *Tpc2* deficient mice were found to display extensive cholesterol accumulation mimicking NAFLD phenotypes, although pH_[lys]_ was unperturbed [84]. It was speculated that calcium flux through TPC2 might aid the fusion of endolysosomal structures.

#### 2.2.5. Store-Operated Calcium Channels (SOCCs)

Store-operated Ca^2+^ entry (SOCE) is an essential mechanism for the maintenance of intracellular Ca^2+^ homeostasis and can be activated upon depletion of cellular Ca^2+^ stores. SOCC are plasma membrane Ca^2+^ influx channels that have a multitude of physiological roles including autophagic regulation. Activation of SOCE by one such plasma membrane SOCC, Orai1, together with its ER-localized interactor protein STIM, has been shown to activate CaN. Activated CaN thereafter activates the canonical TFEB nuclear translocation pathway, aiding transcription of autophagic and lysosomal genes [85]. In an induced hypercholesterolemia model of endothelial progenitor cells, SOCE activation led to decreased cell proliferation but increased autophagic activity. Here, increased intracellular Ca^2+^ was found to activate CAMKKβ and downregulate mTOR [86]. Another study demonstrated that resveratrol-induced SOCE inhibition and concomitant Ca^2+^_[ER]_ depletion leads to unfolded protein response and ER stress, effectively leading to autophagic cell death of cancer cells [87]. In other words, the SOCE contribution to autophagy is also highly context-dependent.

### 2.3. Vacuolar H^+^-ATPase

The vacuolar-type proton-ATPases gain energy from ATP hydrolysis to pump protons across plasma or intracellular membranes to the lumen or intracellular compartments, respectively. V-ATPase is a multi-protein complex composed of two domains: the membrane-peripheral cytosolic V1 domain, which is composed of subunits A–H, and it provides the catalytic function (ATP hydrolysis); and the membrane embedded Vo domain, which is composed of subunits a, d, e, c and c″ (an additional subunit, c′ is present in yeast, and higher eukaryotes also contain an accessory), and Vo provides the trans-membrane proton-pumping function of V-ATPase [88]. ATP hydrolysis and proton transport are coupled via a rotary mechanism in V-ATPase, which ensures that V-ATPase pumps protons across lipid membranes in an ATP-dependent manner [89]. Hence, V-ATPase is a key actor in the regulated acidification of intracellular organelles, such as lysosomes, and the degradative function of acidic hydrolases [90]. In vitro and in vivo studies demonstrated that V-ATPase is also necessary for the mTOR complex 1 to sense lysosomal AA levels. As discussed earlier, Rag GTPases and Ragulator complex provide an AA-sensitive docking site for mTORC1 on the surface of the lysosome. Additionally, V-ATPase was also identified to affect mTORC1 function both in *Drosophila* S2 and in human HEK293T cells. Silencing of V-ATPase subunits (*Drosophila* vhaAC39, vha16, vha100-1, and vha100-2 and human ATP6V0c, the ortholog of *Drosophila* vha16) decreased AA-induced phosphorylation of mTORC1 substrate S6K. Inhibiting V-ATPase by concanamycin A (ConcA) and salicylihalamide A (SalA) showed similar results regarding mTORC1 kinase activity; moreover, these inhibitors blocked mTORC1 recruitment to lysosomes in response to amino acids. These indicate V-ATPase’s role in mTORC1 activation and localization to the lysosomal surface. Different rescue experiments showed that V-ATPase acts downstream of AA, but upstream of Rag GTPases and the authors suggested an “inside out” AA sensing mechanism coming from the lysosome, for which V-ATPase is required. Under physiological conditions, lysosomal V-ATPase activates mTOR complex via Rag GTPases by recruiting mTORC1 to lysosomes. EN6, a small-molecule in vivo activator of autophagy, covalently targets the ATP6V1A subunit of V-ATPase. The modified ATP6V1A decouples the V-ATPase complex from Rags, which leads to mTORC1 inhibition, activation of autophagy and increased lysosomal acidification [91]. Therefore, while generally thought as a downstream component of autophagy, V-ATPase can regulate upstream components of the process. This is most likely related to the ability of mTORC1 to integrate environmental and intracellular signals to coordinate cell growth and metabolic processes.

Besides the role of V-ATPase in lysosomal acidification, it is becoming more and more certain that subunit c and other Vo subunits play direct roles in the vesicle transport processes by facilitating membrane fusion, via intra- and intermembrane subunit rearrangement and interactions with other fusion proteins unrelated to the acidification role of V-ATPase [92,93]. While lysosomal pH was found to be important for autophagosome-lysosome fusion in cultured CHO cells based on neutralizing lysosomes with NH_4_Cl, such treatments can have indirect effects [94]. Indeed, lysosomes remained fusion competent in V-ATPase-deficient tissue that led to formation of giant autolysosomes in *Drosophila* melanogaster. Of note, the commonly used V-ATPase inhibitor bafilomycin A1 is known to block the ER-resident SERCA calcium pump as well, and the inhibition of SERCA (and a concomitant increase in Ca^2+^_[cyt]_) rather than V-ATPase loss was found to perturb autophagosome–lysosome fusion. In line with this, SERCA activation promoted fusion in bafilomycin-treated cells [55]. PSEN2, one of the few proteins found to contribute to early onset familial Alzheimer’s disease, was suggested to impair V-ATPase assembly by preventing glycosylation of the V-ATPase subunit V0a1 [95]. Further studies however, found that ER Ca^2+^ is partially depleted due to SERCA inhibition and ER calcium leakage in *Psen2* mutants [96,97]. Thus, cytosolic and lysosomal Ca^2+^ dyshomeostasis in *Psen2* mutants likely impairs Rab7 recruitment to autophagic vesicles and the degradative capability of lysosomes, leading to fusion and degradation defects [98].

## 3. Regulation of Lysosomal Ion Channels

### 3.1. Regulation by Lipids

#### 3.1.1. PI(3,5)P2 and Sphingomyelin Regulation of TRPML1 Function

The PIKfyve complex phosphorylates PI(3)P to generate PI(3,5)P2 in low abundance but with clear physiological significance in terms of endo-lysosomal function [99]. Notably, PI(3,5)P2, but not the other related phosphoinositide derivatives PI(3)P and PI(5)P, directly and specifically triggers TRPML1 Ca^2+^ efflux [100]. Depletion of PI(3,5)P2 by overexpressing the phosphatase MTMR that dephosphorylates PI(3,5)P2 to PI(5)P, or by PIKfyve silencing, results in autolysosome degradation defects in yeast [101].

**Figure 2:**
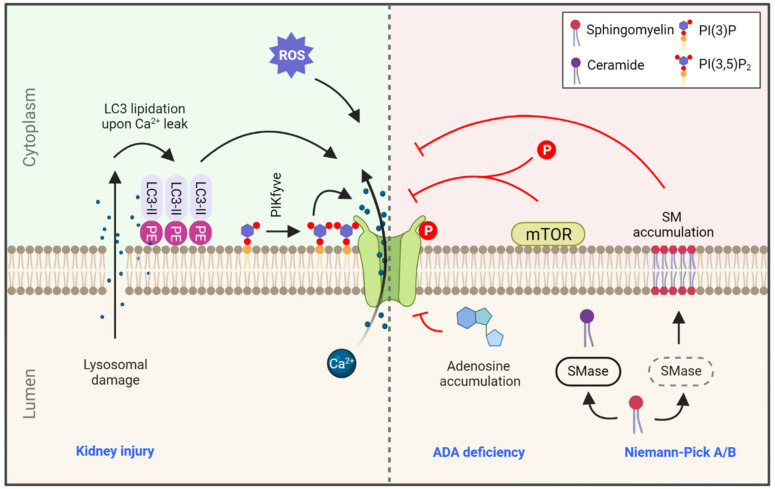
Regulation of TRPML1 in health and disease. TRPML1-mediated calcium efflux is regulated by a variety of signals, such as calcium leakage during lysosomal damage leading to LC3 lipidation at the lysosomal membrane. This lysosome-localized LC3-II is a potent activator of TRPML1. ROS levels and phosphatidylinositol 3,5-bisphosphate (PI(3,5)P2) also positively regulate TRPML1 function. On the contrary, accumulation of adenosine due to ADA deficiency and accumulation of SM in the lysosome due to SMase deficiency in Niemann-Pick type A/B are strong inhibitors of TRPML1 activity. Lastly, TRPML1 is also regulated by mTOR phosphorylation, and consequently by small molecules that regulate mTOR activity (rapalogs; see text). Blue text indicates related diseases. SMase: sphingomyelinase, ADA: adenosine deaminase, PE: phosphatidylethanolamine, PIKfyve: phosphoinositide kinase, FYVE-type zinc finger containing.

TRPML1 is also regulated by SM, but not by related membrane lipids such as ceramide and phosphocholine. This regulation is thought to prevent TRPML activation at undesired cellular locations, such as the plasma membrane, which is rich in SM, instead of the lysosome where SM is rapidly degraded by lysosomal SMase (Figure 2). According to this model, Niemann-Pick type C1 cells that accumulate undegraded SM in lysosomes due to SMase defects might interfere with TRPML function, resulting in undegraded cholesterol accumulation [56,102].

#### 3.1.2. TRPML1 Regulation by Lipidated LC3 upon Injury

A recent study found that the ATG conjugation system that mediates LC3 lipidation is essential for the lysosomal damage response during kidney injury (for example, in nephropathy characterized by deposition of calcium oxalate crystals). Interestingly, lipidated (but not lipidation-deficient) LC3 was shown to co-immunoprecipitate with lysosomal TRPML1. Conversely, TRPML1 activation by ML-SA1 increased lysosome-localized LC3 lipidation. Mechanistically, it was shown that mild Ca^2+^ efflux during lysosomal damage can initiate an unusual lysosomally-localized LC3 conjugation, and this is followed by the TRPML1-LC3 interaction that enhances TRPML1 Ca^2+^ efflux following injury. This Ca^2+^ efflux promotes canonical TFEB activation towards transcriptional upregulation of lysosomal genes [103].

### 3.2. Regulation by mTOR and Calcium

Studies in *Drosophila* suggested that TRPML may be required for autophagosome/endosome/amphisome-lysosome fusion in flies, and it regulates TOR kinase, as TRPML deficient cells showed decreased TOR activity [71]. Conversely, enhanced TOR activity promoted plasma membrane localization of TRPML, which decreased the presence of TRPML on late endosomes and hence fusion of late endosomes with lysosomes. In another study genetic interaction was found between TRPML and AMPK in *Drosophila* [104]. It was therefore hypothesized that the regulation of this ion channel occurs through phosphorylation of TRPML1 by AMPK. In addition, immunoprecipitation and mass spectrometry data revealed that TRPML1 interacts with mTOR, which phosphorylates TRPML1 on its C-terminal serine residues (Ser572, Ser576), which results in decreased channel activity [104]. Additionally, a reciprocal feedback loop of TRPML1-mTOR-CaM was identified in mammalian cells by two research groups [66,69]. Proper Ca^2+^ release through TRPML1 ensures binding of CaM to mTOR, which facilitates its lysosomal recruitment and kinase activity, and hence deactivates the channel. In human fibroblasts this feedback loop was proved to be essential in ALR, in which nascent lysosomes are re-generated after degradation is completed in autolysosomes [66].

TPC1 and TPC2 were identified as ATP-sensitive Na^+^ channels (lysoNaATP) that detect nutrient status together with mTORC1. Knockdown and overexpression studies of mTOR revealed that mTORC1 is responsible for the ATP- and rapamycin-sensitivity of lysoNaATP, as the channel was inhibited for longer by ATP when mTORC1 was released from lysosomes during nutrient deprivation [105]. Alternatively, if cellular ATP level decreases, mTORC1 dissociates from lysosomes (and TPCs), which leads to opening of the channel. In the same cell type, TPC2-overexpressing cells produced concentration-dependent Ca^2+^-signals after treatment with different amounts of rapamycin (note that wild-type HEK cells express low levels of TPC2). In line with this, the mTORC1 inhibitors rapamycin and torin-2 did not induce measurable Ca^2+^ transients in *Tpc2* KO mouse myocytes [79]. Conversely, TPC2 can somehow affect mTORC1 activity, as reduced levels of phosphorylated mTORC1 substrate phospho-S6K and its target phospho-S6 ribosomal protein were detected in cultured myotubes derived from *Tpc2* KO mice. Furthermore, the reactivation of mTORC1 after starvation was delayed, and hence autophagy termination was also impaired [22].

TMBIM6 is a Ca^2+^ channel-like protein in the ER membrane and TMBIM6-associated Ca^2+^ release was shown to increase lysosomal Ca^2+^ level in human fibrosarcoma cells (HT1080). Based on proximity-ligation assay, it was shown that TMBIM6 specifically localizes to ER-lysosome contact sites. Additionally, ML-SA1-induced Ca^2+^ efflux from lysosomes was lower in TMBIM6-silenced cells than in controls, which suggests that TMBIM6-associated Ca^2+^ leakage increases TRPML1-mediated Ca^2+^ release from the lysosomes [106].

### 3.3. Regulation by Small Molecules

Regulation of TRPML1 by ROS proved to be a key link between ROS and autophagy [75]. Elevation of mitochondrial ROS (mtROS) levels or exogenous oxidants activates TRPML1, leading to Ca^2+^ release. This activation will promote translocation of TFEB into the nucleus in a CaN-dependent manner, where TFEB induces autophagy and lysosome biogenesis [70]. The activation of TRPML1 was investigated using different cell types and agents. In COS-1 cells, TRPML1 was strongly activated after the administration of ChT, a non-selective strong oxidant, along with other oxidants such as N-chlorosuccinimide, NaOCl, thimerosal, t-butyl hydroperoxide (TBHP) and H_2_O_2_ (although less potently), with similar effects across cell types. Additionally, in *Trpml1* KO mouse macrophages, no ChT-activated inward currents could be detected, which suggests that TRPML1 is a major ROS-regulated channel in the lysosome [75]. In another study, the plant alkaloid lycorine was investigated, which was found to decrease the level of mtROS in osteoclasts and attenuate autophagy via the mtROS-TRPML1-TFEB axis. Thus, lycorine was suggested to protect against LPS-induced bone loss in mice [107]. TRPML1 was found to be regulated not just by mTORC1, but by the mTORC1 inhibitor rapamycin as well [108]. Rapamycin strongly activated TRPML1 based on whole-endolysosome recordings performed on vesicles isolated from EGFP-TRPML1-transfected COS1 cells and in wild type parietal cells. This effect, however, proved to be mTOR independent, since torin-1, a catalytic mTOR inhibitor, could not activate TRPML1. Moreover, the effects of other commercially available mTOR-inhibiting rapamycin derivatives (rapalogs) seemed to be inconsistent: Tem and Eve activated TRPML1, and no activation was seen for Defo or Zota. This difference among rapalogs suggests that the activating effects of rapamycin, Tem and Eve on TRPML1 are independent of mTORC1 inhibition. Downstream effects of TRPML1 activation and Ca^2+^ release were also investigated in the study: rapamycin and Tem induced TFEB nuclear translocation in TRPML1-overexpressing HeLa cells and thus increased autophagic flux [108].

Alteration of TRPML1 activity is a potential therapeutic strategy in diseases connected to adenosine dyshomeostasis, with which elevated levels of intracellular adenosine can be found, such as ADA (lymphopenia, SCID) or ENT3 deficits (familial Rosai-Dorfman disease, H syndrome and Faisalabad histiocytosis). ADA deficiency elevates the lysosomal adenosine level, which inhibits TRPML1 (Figure 2). ADA-deficient human fibroblasts showed enlarged, alkalinized lysosomes with decreased proteolytic activation due to an elevated adenosine level. These effects could be rescued via TRPML1 activation by ML-SA1 [109].

While ML-SA1 is widely used experimentally to alter TRPML1, it is not selective for TRPML1 isoforms [56]. Besides ML-SA1, many TRPML agonists have been developed, and isoform-selective activators also exist, such as ML2-SA (selective for TRPML2) [110] and SN-2 and EVP-21 (selective for TRPML3) [110,111]. However, neither TRPML1 selective agonists nor TRPML1 selective inhibitors could be developed initially (only antagonists without isoform selectivity are available: ML-SI1 and ML-SI3). Recently, the estradiol analogs 17β-estradiol methyl ether (EDME), PRU-10 and PRU-12 were identified as selective TRPML1 antagonists. These estradiol analogs effectively inhibit Ca^2+^ release through TRPML1 channel, nuclear translocation of TFEB and autophagy, though their effect on the estradiol receptor is low [76].

A potent activator of lysosomal Ca^2+^ release is NAADP [112]. TPC2 was suggested as a candidate that is activated by NAADP, based on the inhibition of TPC2 expression using siRNA [113] and KO mice [114], which resulted in decreased NAADP-evoked Ca^2+^ release. However, using photoaffinity labelling (utilizing a radioactive photoprobe, which binds to NAADP receptors with high affinity), TPC2 could not be identified as a NAADP receptor. It was instead suggested that NAADP-gated channels (TPC) and NAADP receptors are different molecular entities, and NAADP binds to an adaptor protein in a larger TPC complex [115]. In another study, TPC2 was found to be activated by NAADP in the absence of Mg^2+^ (an inhibitor of TPC2). Additionally, JNK and p38 kinases inhibit TPC2 in HEK293 cells [116]. Finally, glutamate was found to induce autophagy through the NAADP-TPC-AMPK axis in SHSY5Y neuroblastoma cells [117].

## 4. Interrelationship between Calcium and V-ATPase during Autophagy

As TRPML appears important to proper lysosomal function we focus on the possible role of Ca^2+^ in regulating V-ATPase function below. As discussed earlier, the proton translocating V-ATPase ensures acidic lysosomal pH, and it is also necessary to some other vesicular organelle functions. It consists of two multiprotein subunits, of which the membrane-associated V0 multiprotein complex is responsible for conducting protons into the lumen, and the V1 complex on the cytoplasmic side of the membrane forms the ATPase part of the holoenzyme [118,119]. Proper functioning of the holoenzyme is required for the acidification of various organelles, such as the lysosome, endosome, dense core and synaptic vesicles [120,121]. The overall structure of the holoenzyme resembles that of the F-ATPase/synthase, which catalyzes a reaction opposite to that of V-ATPase [122]. Some subunits are represented by multiple paralogs even in yeast but particularly in higher eukaryotes [123]. Therefore, various V-ATPase assemblies can form, whose intracellular or organ-specific distributions are dictated by their compositions [124].

The establishment and maintenance of a proton gradient is vastly ATP consuming, so V-ATPase function is regulated to perform optimally in the most appropriate time and context. V-ATPase activity depends on the active participation of protein complexes such as RAVE in yeast and rabconnectin-3 complexes in higher eukaryotes whose function is to catalyze the assembly of V1 and V0 sectors [125,126,127,128].

The most obvious candidate for a Ca^2+^ regulated membrane process is vesicle fusion mediated by SNAREs. While readers can find excellent reviews on this topic, we cannot omit their mentioning entirely, as SNARE function seems to be regulated by V-ATPase, at least in some contexts. The neuronal specific V0 subunit a1 (V100 in *Drosophila*) was shown to interact with calmodulin in a Ca^2+^-dependent manner in *Drosophila*, and mutations compromising its CaM binding interfered with synaptic vesicle function [129,130]. Supporting a possible role of V-ATPase in SNARE mediated membrane fusions, interactions were found between the V0 sector and various SNAREs, including VAMP2, syntaxin 1A, SNAP25 and syntaxin 17, in a V0 ’d’ isoform-specific manner in the latter case [129,131,132]. In vitro, V100 in the absence of Ca^2+^-CaM disrupts t-SNARE assembly, which, on the other hand, is facilitated by the presence of Ca^2+^-CaM [133]. Interestingly, the *Drosophila* V0 subunit a1 can complement the loss of its *S. cerevisiae* homolog regarding the vacuolar fusion phenotype of yeasts, but not the acidification defect [129]. This probably means that the acidification and membrane fusion functions of V-ATPase are separable processes, so V-ATPase may function in fusion independently from its H^+^ translocating ability [55]. It is important to emphasize that the role of Ca^2+^-CaM and V-ATPase interaction was only demonstrated in the context of the neuronal specific V0a1, though.

There are numerous other examples for links between V-ATPase and Ca^2+^ signaling. Rabconnectin 3 appears to be closely linked to and can alter the function of the voltage gated calcium channel Cav2.2 [134]. Interestingly, another voltage gated calcium channel Cav2.3 has been shown to directly bind to the G1 subunit of V-ATPase [135]. Rabconnectin 3 also interacts with the calcium dependent protein CAPS1 (also known as; CADPS), a known binding partner of SNAREs [136]. Rbcn (Rbcn-3A and Rbcn-3B) in *Drosophila* are required for Notch signaling, and unprocessed Notch accumulates in late endosomal compartments in their absence [137]. Mutations in *VhaAC39*, the gene encoding one of the two paralogues of the’d‘ subunit of the V0 sector of V-ATPase, phenocopied *Rbcn-3A* and *Rbcn-3B* mutants [137]. Most notably, *Rbcn-3A* and *Rbcn-3B* mutant cells lack acidic compartments, similarly to *VhaAC39* mutant cells [137]. Notch signaling is also compromised by loss of Rabenosin function and disrupted V-ATPase activity in mammalian cells [138]. These findings were partly recapitulated in the context of dense core vesicle acidification in neurons, where not only Rbcn but also CAPS1 proved to be critical for vesicle acidification [136]. Thus, CAPS1 may relay calcium signals to regulate V-ATPase assembly by Rabconnectin-3.

Isolated and lipid bilayer reconstituted V0 c subunits rings of V-ATPase from *S. cerevisiae* form dimers and can act as large-conductance transmembrane channels [139]. Earlier studies characterized a vacuolar channel from yeast whose opening is regulated by membrane potential and the presence of Ca^2+^ at the outer (cytoplasmic) side of the membrane [140]. Remarkably, F-ATP synthase can form a Ca^2+^ dependent high-conductance channel, which resembles the mitochondrial permeability transition pore [141]. Since knowledge concerning F-ATP synthase has been instrumental for understanding V-ATPase function in the past, it is tempting to speculate that the potential Ca^2+^ dependency of the vacuolar channel may be related to a putative capability of the V0c subunit to bind Ca^2+^.

The activity of V-ATPase (purified from clathrin-coated vesicles observed during synaptic vesicle maturation) has been shown to be supported by either Mg^2+^ or Ca^2+^ [142]. However, unlike Mg^2+^, Ca^2+^ seemed to support only ATP hydrolysis without vectoral proton pumping, as in the case of F-ATP synthase [143]. A possible explanation for this finding is the dual role of divalent cations during the operation of the holocomplex: on one hand, these may support ATP hydrolysis, while on the other hand they may have a structural role to facilitate proper subunit arrangement and ensure the coupling of ATP consumption and proton translocation [142]. Most remarkably, however, it was shown later that Ca^2+^ can support a coupled ATPase-H^+^ transfer reaction depending on two critical conditions, one being that V-ATPase is incorporated into liposomes, and the other being that there is a favorable membrane potential difference between the intra- and extraliposomal space [144]. Such a membrane potential difference surely exists in the context of both lysosomes and synaptic vesicles, and it is one of the driving forces for neurotransmitter uptake in the latter case, pointing toward the physiological relevance of the above observation [28,145,146]. Considering that loss of TRPML1 perturbs lysosome acidification, a direct role of Ca^2+^ released from lysosomes in triggering V-ATPase proton translocation could be proposed [147]. Importantly, Ca^2+^ accumulation in synaptic vesicles partly depends on the counter transport of H^+^ and Ca^2+^ and might be secondary to acidification [148]. However, most data on V-ATPase function are based on observations using complexes purified from synaptic vesicles and not from lysosomes, so the direct applicability of these findings in the context of autophagy is unclear.

## 5. Lysosomal Ion Channels in Health and Disease

Lysosome-associated ion channels such as TRPML1, TPCN and TMEM175 have been connected to neurodegenerative diseases, such as Parkinson’s disease (PD) and Alzheimer’s disease [38,49,149]. Of these channel proteins, TRPML1 is by far the most studied from the perspective of pathological mechanism and therapeutic potential. Mutations in TRPML1 result in MLIV, a neurodegenerative disease first recognized in 1974 [150], with phenotypes including mental disability, motoric dysfunction, retinal degeneration and shortened lifespan [151]. On the cellular level, the mutation of TRPML1 causes enlarged late endosomes and lysosomes, indicating the impairment of lysosomal degradation and retrograde lipid transport [56]. Aside from its loss-of-function, multiple recent studies also connect enhanced TRPML1 activity with the progression and malignancy of various cancer types, including melanoma, glioblastoma and non-small-cell lung carcinoma [152,153,154]. A common thread across these studies is the increased autophagic activity partially supported by TRPML1, which in turn enhances the survival of malignant cells.

Recently, novel physiological roles of TRPML1 were uncovered. By examining the differentiation of bone marrow stroma-derived OP9 cells into adipocytes, a study established the gradual upregulation of TRPML1 in the differentiating cells that coincided with the increase of the adipocyte marker PPARγ [155]. Importantly, the authors found that the siRNA-mediated suppression of TRPML1 inhibited adipocyte differentiation and the release of lysosome-derived exosomes [155]. TRPML1 was also found to control gastric acid secretion in mice. The mutant mice suffered from hypochlorhydria, decreased secretion of gastric acid under normal and histamine-induced conditions, resulting in higher, more alkaline, gastric pH. Further analysis of the phenotypes revealed that *TRPML1* mutant parietal gland cells harbored large LAMP1-positive vacuoles that also accumulated mislocalized foci of H^+/^K^+^ ATPase, and were unable to fuse with the secretory membranes of the gastric parietal cells and empty their contents towards the luminal side [156].

Importantly, lysosomal ion channels are also involved in the pathogenesis of infectious diseases. Recently, a study reported that *Helicobacter pylori* can utilize its vacuolating toxin A (VacA) to directly inhibit TRPML1 activity and shelter itself intracellularly in a protective vacuole in response to antibacterial therapy [157]. As an underlying mechanism, the authors showed that VacA-induced TRPML1 dysfunction causes MLIV-like defects in autophagosome-lysosome fusion, which is confirmed by LC3-II accumulation. They also showed that overexpression and agonist-mediated activation of TRPML1 inhibit *H. pylori* reservoir formation, restoring functional autophagy and bacterial killing in the infected cells [157]. However, the therapeutic potential of TRPML agonists such as ML-SA1 was investigated not only against bacteria, but viruses as well. In another study, ML-SA1 was used to activate TRPML1 channels in the context of DENV2 and ZIKV infections in Huh7, A549 and HEK293T cell lines. Interestingly, TRPML1 activation decreased viral titers for both DENV2 and ZIKV through enhancing lysosomal acidification and endosome–lysosome fusion [147]. The authors also found that ML-SA1 can suppress the DENV2 infection even if the more upstream autophagic machinery is compromised, suggesting that this is an autophagy-independent process.

Melastatin-type TRP channel TRPM2 was recently shown to undertake similar roles to TRPML1 in mASMs. *TRPM2* mutant mASMs were unable to efficiently complete autophagy due to inhibited transition of autophagosomes into autolysosomes and impaired autolysosomal degradation. Furthermore, it was demonstrated that TRPM2 was responsible for the H_2_O_2_-induced rise in cytosolic Ca^2+^, at least partially via the mobilization of Ca^2+^ from lysosomes. Ultimately, the loss of TRPM2 was found to interfere with the starvation-induced autolysosomal acidification and degradation process and to partially inhibit autophagy-induced cell death [158]. In a similar way, defective function of the lysosome-bound TMEM175 K^+^ channel was associated with altered lysosomal pH and enzyme activity, causing the accumulation of phosphorylated α-synuclein, potentially contributing to the development of PD [38,149].

With these recent advances, it is becoming more evident that lysosomal ion channels should be considered as therapeutic targets in lysosomal storage diseases, in malignant cells that survive by inhibiting autophagy-induced cell death and in infectious diseases that compromise lysosomal function. In fact, a growing number of research groups are exploring the possibility of targeting key lysosomal ion channels such as TRPML1 and TMEM175 in neurodegenerative diseases [74,149,153].

## 6. Conclusions and Perspectives

In this review, we aimed to discuss current knowledge of how distinct steps of the autophagy-lysosomal process are regulated by ion transporters (Figure 1). For brevity, we mostly excluded tissue-specific ion channels such as the plasma membrane CFTR or ER-localized RyR, which are reported to influence autophagy in specific tissue types [159,160]. A few regulatory nodes become clear if we look at ion channel regulation of autophagy (summarized in Figure 1). First, activation of autophagosome biogenesis by CaM-CAMKKβ is a recurring theme under regulation by lysosomal, ER and plasma membrane Ca^2+^ channels. Next, the CaN-TFEB transcriptional regulation of autophagy and lysosomal biogenesis genes is a critical mechanism that is intrinsically tied to mTORC1, but it has another layer of regulation by factors independent of nutrient status (such as cellular ROS burden [75]). Another key point of regulation seems to be ER-lysosomal Ca^2+^ transport by the ER Ca^2+^ channels IP_3_R, RyR and TMBIM6 at contact sites between these organelles. Since lysosomes (and mitochondria) depend on the ER for Ca^2+^ replenishment, any calcium homeostasis defect in the ER has adverse effects on degradation of autophagic cargo. We conclude by highlighting two important directions for research:

- It appears that defects of ion homeostasis either in the lysosome or in cytosol influence autophagic flux. The multitude of roles of TRPML1 is especially surprising in this regard: Ca^2+^ and Zn^2+^ transport by TRPML1 seem to have opposite effects on autophagic progression. This might indicate that Ca^2+^ and Zn^2+^ efflux by TRPML1 is context-sensitive—e.g., zinc efflux being prominent in proliferating tumor cells vs. calcium efflux being required for completion of basal autophagy. Additionally, TRPML1 functions as a H^+^ leaking channel to maintain physiological pH_[lys]_, and absence of this proton permeability function likely contributes to over-acidified autolysosomes containing undegraded cargo in *Trpml* mutant fly tissue. Whether this proton leakage is coordinated with V-ATPase function during autophagy is an intriguing question, and it could represent a Ca^2+^ independent layer of V-ATPase regulation by TRPML1.
- ER-lysosome contact sites are especially important for lysosomal calcium replenishment. How dynamic these contact sites are during nutrient replete versus nutrient depleted conditions remains to be established. Additionally, it will be important to study the relationship of ER microdomains and exit sites (which are often sites of autophagosome biogenesis, [161]) during autophagosome formation, ER-lysosome contact site maintenance and autophagosome-lysosome fusion events.

## Data Availability

Not applicable.

## Abbreviations

AA	amino acid
ADA	Adenosine deaminase
AKT	three serine/threonine-specific protein kinases
ALG-2	apoptosis-linked gene-2 protein
ALR	aumiddlehagic lysosome reformation
ALSAM2	amyotrophic lateral sclerosisM2 proton channel, influenza virus A
AMPK	AMP-activated protein kinase
Arl8	Arf like protein 8
ATF6	activating transcription factor 6
Atg	aumiddlehagy genes
ATP	adenosine triphosphate
CaM	calmodulin
CAMKKβ	Ca^2+^/calmodulin dependent protein kinase kinase-beta
CaN	calcineurin
CAPS1/CADPS	calcium dependent activator protein for secretion
Cav2.2	(N-type) voltage-gated Ca^2+^ channels
CFTR	cystic fibrosis transmembrane conductance regulator
ChT	chloramine T
CLA	clarithromycin
CPP	calcium phosphate precipitate
DENV2	dengue-virus, strain 2
DEPTOR	DEP- domain-containing mTOR- interacting protein
DFCP1	double FYVe domain-containing protein 1
EDME	estradiol methyl ether
ENT3	equilibrative nucleoside transporter 3
ER	endoplasmic reticulum
ERK1/2	extracellular signal-regulated protein kinase
F-ATPase	F-type ATPase/ATP synthase
FIP200	FAK family–interacting protein of 200 kD
FOXO3	forkhead box protein O3
GRP75	glucose-regulated protein 75
GTPase	guanosine triphosphate enzyme
H2O2	hydrogen peroxide
hERG1	Ether-A-Go-Go-Related Gene Potassium Channel 1
HOPS	homotypic fusion and vacuole protein sorting
IP3R	inositol trisphosphate receptor
IRE1	inositol-requiring enzyme 1
JNK	c-Jun N-terminal kinases
KEL	K^+^-selective channel
KO	knockout
Kv11.3	pore-forming (alpha) subunit of voltage-gated potassium channel
L-BMAA	neurotoxin β-N-methylamino-L-alanine
LC3	microtubule-associated light chain-3
LPS	lipopolysaccharide
mASMs	mouse aortic smooth muscle cells
MCOLN1	mucolipin-1
ML-SA1	mucolipin specific agonist, 1
ML2-SA	mucolipin2 specific agonist
MLIV	mucolipidosis type IV
mLST8	mammalian lethal with SEC13 protein 8
MTMRs	myotubularin-related proteins
mTOR	mechanistic target of rapamycin
mtROS	mitochondrial reactive oxygen species
NAADP	nicotinic acid adenine dinucleotide phosphate
NH4Cl	ammonium chloride
ORAI1	calcium release-activated calcium channel protein 1
p38	mitogen-activated protein kinase p38 beta
p62	sequestosome-1
PE	phosphatidylethanolamine
PERK	protein kinase (PKR)-like ER kinase
PI(3,5)P2	phosphatidylinositol 3,5-bisphosphate
PI3K-III	class III phosphatidylinositol 3-kinase complex
PI3P	phosphatidylinositol 3-phosphate
PIKFYVE	phosphoinositide kinase, FYVE-Type Zinc Finger Containing
PRAS40	raptor recruits proline-rich AKT substrate 40 kDa
PROTOR1/2	protein associated with rictor 1 or 2
PSEN2	presenilin 2
Rab7	Ras-related protein7
Raptor	regulatory-associated protein of mTOR
RAVE	regulator of ATPase of vacuoles and endosomes
Rbcn	rabconnectins
ROS	reactive oxygen species
RYR	ryanodine receptor
SCID	severe combined immunodeficiency
SERCA	sarco/endoplasmic reticulum calcium ATPase
SM	sphingomyelin
SMase	sphingomelinase
SNAP25	synaptosomal-associated protein 25
SNAP29	synaptosomal-associated protein 29
SNARE	soluble N-ethylmaleimide-sensitive factor attachment protein receptor
SOCC	store-operated calcium channels
SOCE	store-operated Ca^2+^ entry
STIM1	stromal interaction molecule 1 precursor
TBHP	t-butyl hydroperoxide
TFEB	transcription factor EB
TG	thapsigargin
TMBIM6	transmembrane Bax inhibitor motif-containing 6
TMEM175	transmembrane protein 175
TOM70	component of the TOM (translocase of outer membrane) complex
TPC2	two pore channel 2
CAX2	vacuolar cation/proton exchanger 2
TRPM2	transient receptor potential cation channel subfamily M member 2
TRPML	transient receptor potential mucolipin channel
TSC2	tuberous sclerosis complex-2/tuberin
ULK1	unc-51 like aumiddlehagy activating kinase 1
V-ATPase	vacuolar-ATPase
VacA	vacuolating toxin a
VAMP2	synaptobrevin-2/ vesicle-associated membrane protein 2
VAMP7	vesicle-associated membrane protein 7
VAMP8	vesicle-associated membrane protein 8
VDAC1	voltage-dependent anion-selective channel protein 1
VhaAC39-1	*Drosophila melanogaster* vacuolar H^+^ ATPase AC39 subunit 1
VMP1	vacuolar membrane protein-1
VPS3	vacuolar protein sorting 34
ZIKV	Zika-virus
